# The Application of Exercise Training for Diabetic Peripheral Neuropathy

**DOI:** 10.3390/jcm10215042

**Published:** 2021-10-28

**Authors:** Clifton J. Holmes, Mary K. Hastings

**Affiliations:** 1 Program in Physical Therapy, Washington University School of Medicine, St. Louis, MO 63110, USA; hastingsmk@wustl.edu; 2 Center for Human Nutrition, Washington University School of Medicine, St. Louis, MO 63110, USA

**Keywords:** diabetes mellitus, sensorimotor, physical training, therapy, hyperglycemia

## Abstract

Diabetic peripheral neuropathy (DPN) is the presence of symptoms and/or signs of peripheral nerve dysfunction in people with diabetes after the exclusion of other causes. It is associated with pain, paresthesia, sensory loss, muscle atrophy with fat infiltration, and muscular dysfunction typically starting distally in the feet and progressing proximally. Muscle deterioration within the leg and foot can lead to muscle dysfunction, reduced mobility, and increases the risk of disability, ulceration, and amputation. Exercise training is an established method for increasing the different components of physical fitness, including enhancing body composition and improving neuromuscular strength. A number of experimental studies have utilized exercise training to treat various impairments associated with DPN, such as nerve conduction velocity, pain tolerance, and balance. However, the broad spectrum of exercise training modalities implemented and differences in target outcome measurements have made it difficult to understand the efficacy of exercise training interventions or provide appropriate exercise prescription recommendations. Therefore, the aims of this review were to (1) briefly describe the pathophysiology of DPN and (2) discuss the effects of exercise training interventions on sensorimotor, metabolic, and physical functions in people with DPN.

## 1. Introduction

Diabetes mellitus remains one of the largest metabolic diseases in the world, having high prevalence in both developed and developing countries. As of 2019, approximately 463 million adults between the ages of 20–79 were living with diabetes across the globe while 4.2 million deaths had occurred as a result of diabetes. Moreover, an estimated 1 in 5 adults with diabetes are over the age of 65 [1]. According to the latest National Diabetes Statistics Report, an estimated 34.2 million people across all ages had diabetes in the US population [2]. Chronic diabetes can cause a series of nerve, blood vessel, and lymph vessel disorders, such as radiculopathy, plexopathy, and mononeuropathy [3]. However, the most common complication of diabetes is the emergence of diabetic peripheral neuropathy (DPN), which affects the somatic sensory and motor nerves and autonomic nerves [4]. Defined as “the presence of symptoms and/or signs of peripheral nerve dysfunction in diabetics after the exclusion of other causes”, DPN is damage to the sensory and motor nerves of the feet, legs, hands, and arms in response to long-term elevation of blood glucose levels. It is associated with pain, paresthesia, sensory loss, muscle atrophy with fat infiltration, and muscular dysfunction in the limbs [5]. According to a position statement by the American Diabetes Association, the prevalence of DPN in US adults is an estimated 28% [6]. A key point of concern among those with DPN is the acceleration of deterioration of leg and foot musculature. Muscle dysfunction in the calf and foot contributes to foot deformity, which in turn increases the risk of disability, ulceration, and amputation [7]. Moreover, muscle dysfunction in the lower extremities through damage of the sensory and motor nerves can severely impair mobility, which is of even greater concern for older adults already at an elevated risk of falling. It has been hypothesized that improvement in both balance and stability, as well as strengthening of the lower-body of individuals with DPN, has the potential to enhance gait and physical capacity, while also reducing disability, foot deformity, and the risk of amputation [8,9,10].

A muscle’s ability to respond to an exercise stimulus is likely related to the distal to proximal progression of vascular and nervous system disease associated with DPN. The vascular and nervous system mediators of neuropathic muscle adaptation that may be critical are muscle specific microvascular perfusion, glucose uptake, and neural drive. However, the current standard measures of these mediators are not precise or muscle specific. For example, (1) microvascular dysfunction from type 2 diabetes-associated peripheral neuropathy is not detected with common macrovascular assessments (ankle brachial index); (2) muscle specific metabolic function is not adequately measured with whole body indices (homeostatic model assessment of insulin resistance score); and (3) muscle strength, a measure of motor neuropathy, is unable to decouple neural transmission from a muscle’s intrinsic contractility. Different forms of exercise training, including traditional and circuit-based resistance training, have been shown to increase the aforementioned mediators in older adults with and without diabetes, but research done specifically on DPN patients is lacking [11,12,13,14,15,16,17]. The purpose of this review is to (1) briefly describe the pathophysiology of DPN and (2) discuss the effects of exercise training interventions on sensorimotor, metabolic, and physical functions in people with DPN.

## 2. Pathophysiology

Diabetic peripheral neuropathy results from various biochemical perturbations and is categorized by widespread damage to the peripheral nerve fibers, which can lead to pain, foot ulcers, diminished mobility, impaired quality of life, and significant morbidity. The exact mechanisms behind the pathogenesis of DPN are still unknown due to the multifactorial nature of the disease; however, chronic hyperglycemia with a significant drop in insulin sensitivity seems to be at the forefront of DPN causes [18]. Muscle specific glucose uptake, microvascular perfusion, and neural drive seem to be key components in the development of DPN and important mediators of the response to exercise interventions among those with type 2 diabetes (T2D).

A review by Schreiber et al. [19] describes several associations with DPN, including polyol pathway hyperactivity, oxidative and nitrosative stress, and microvascular changes. A chronic hyperglycemic state sets off a cascade of cellular problems including hyperactivity of the polyol pathway, which has been hypothesized to cause excessive oxidative and nitrosative stress leading to the formation of reactive oxygen and nitrogen species, further damaging to neurons [20,21,22]. With rises in insulin resistance, blood glucose levels remain consistently elevated and gradually set off a cascade of physiological effects resulting in damage to the peripheral nerves and blood vessels.

As hyperglycemia persists and further damage accumulates, dysfunction of the microvasculature (i.e., arterioles, capillaries, and venules) becomes a greater concern. The downregulation of glucose transport induces microvascular endothelial dysfunction, resulting in decreased nitric oxide availability, increased permeability, reactive oxygen species, and leukocyte adhesion [18]. As microvascular dysfunction worsens, insulin resistance increases, leading to greater hyperglycemic responses, and thus the cycle continues [18]. Moreover, hyperglycemia is associated with nerve hypoxia, which can produce electrical instability and disrupt the flow of action potentials. This nerve ischemia is especially prevalent in sensory nerves, which results in reductions in intraepidermal nerve fiber density [23,24,25]. Due to declines in nerve perfusion, the long sensory axons in the distal portions of the limbs are the first to succumb to building damage resulting in unacknowledged injuries and wounds in the lower extremity [26]. Motor neurons are also effected, with reductions in neural drive noted by loss of motor nerve conduction velocity, action potential amplitude, and motor units [27]. Eventually, the assault on the motor neurons and overall muscle activation results in physical disabilities of patients with DPN. When left unchecked and untreated, more intense clinical concerns can arise, one of the most common being diabetic foot syndrome, where patients can experience foot ulceration, infection, and potential surgical intervention.

## 3. Methodological Approach

For this article, “exercise” will be defined as “physical activity that is planned, structured, repetitive, and purposive in the sense that improvement or maintenance of one or more components of physical fitness is an objective” [28,29]. Moreover, “physical fitness” will be defined as “the ability to carry out daily tasks with vigor and alertness, without undue fatigue and with ample energy to enjoy leisure-time pursuits and to meet unforeseen emergencies” [28,29]. The health-related components of physical fitness are (1) cardiorespiratory endurance, (2) muscular endurance, (3) muscular strength, (4) body composition, and (5) flexibility [28,29]. Physical fitness is further expanded to include the six skill-related components (i.e., agility, balance, coordination, power, reaction time, and speed) typically associated with athletes and sport performance; however, due to the population being discussed in the current article having compromised physical abilities and many of the skill-related components being outcome measures of the reviewed studies, the authors believed it was prudent to include them.

The authors searched four databases, CINAHL Plus, Google Scholar, PubMed, and Science Direct, for relevant literature, with the last search taking place on 21 June 2021. Additional articles were extracted from the references of the resulting studies. Search terms included “diabetic peripheral neuropathy” (related terms: DPN, neuropathy) and “exercise” (related terms: exercise therapy, exercise training, physical training). Inclusion criteria for the review included full-text articles in English, experimental clinical trials with quantitative analysis, and interventions must be ≥4 weeks. Studies were excluded if they did not include patients with DPN, were observational in nature, or exercise (or exercise-based training or therapy) was not the primary experimental intervention modality with at least one group of subjects with DPN participating in the intervention. Of the 264 initially searched articles, a final total of 41 studies were included in the current review and can be seen in Table 1.

## 4. Exercise-Based Interventions

### 4.1. Mobility and Functional Movement-Based Exercise Training

For many years, the idea of prescribing weight-bearing exercise was not generally accepted, with practitioners believing it to be unsafe for individuals with DPN. Mueller et al. [8] had 29 DPN patients complete either weight-bearing or non-weight-bearing interventions consisting of progressive balance, flexibility, strengthening, and aerobic exercises occurring 3 times a week for 12 weeks. The weight-bearing group showed greater improvements in 6-min walk distance (378 ± 72 to 404 ± 78 m) and average daily step count (4909 ± 1398 to 5593 ± 1449 steps) without an increase in adverse events and the non-weight-bearing group demonstrated greater improvements in HbA1c (7.8 ± 2.1 to 7.4 ± 1.6%) [8]. Following these promising results, El-Refay and Ali [30] had 15 individuals with DPN complete an 8-week program of range of motion, muscle strengthening, balance, and gait training exercises done three times per week. The intervention program significantly increased walking velocity (0.71 ± 0.07 to 0.87 ± 0.18 m/s), reduced step time (0.86 ± 0.17 to 0.63 ± 0.19), and improved ankle range of motion during the stance phase of gait (19.38 ± 2.39 to 20.45 ± 2.16 degrees) [30].

Not all studies initiating these types of interventions produce the desired results. Sartor et al. [10] had 26 individuals with DPN complete 40–60 min therapeutic sessions, twice a week, for 12 weeks. The intervention consisted of a combination of flexibility, muscle strengthening, and functional training geared toward improving the foot rollover process during gait. There were no significant changes observed in plantar pressure under the six foot areas, plantar pressure distribution, or the functional condition of the foot–ankle complex [10]. Lastly, Kanchanasamut and Pensri [31] had 11 DPN patients complete an 8-week home weight-bearing exercise program using a mini-trampoline. There was a trend for peak plantar pressure at the medial forefoot to decrease in the exercise group, but not in the control group [31].

### 4.2. Aerobic Exercise Training

Aerobic exercise is one of the most studied exercise modalities. It recruits large muscle groups to perform dynamic, rhythmic movements done over a prolonged period of time (e.g., walking, jogging, cycling, and swimming). Engagement in regular aerobic exercise sessions is typically done to improve endurance; however, it produces a wide range of additional health benefits. Increases in cardiorespiratory fitness is largely associated with a reduction in all-cause mortality, specifically from cardiovascular disease, and those with high levels of cardiorespiratory fitness have a greater level of habitual physical activity [68,69,70,71,72]. Previous research investigating the effects of aerobic exercise intervention among individuals with T2D has observed many favorable changes in metabolic health, body composition, and maximal oxygen consumption [73,74]. Additionally, meta-analytic data has shown that chronic aerobic exercise can result in significant decreases in HbA_1c_, fasting blood glucose, low-density lipoproteins, triglycerides, systolic and diastolic blood pressure, and body fat percentage [75]. Though evidence exists demonstrating the efficacy of aerobic exercise to help treat metabolic symptoms of type 2 diabetes, it is still necessary to determine the effects this modality of exercise has on peripheral neuropathy and the muscle tissue-mediators associated with DPN.

#### 4.2.1. Sensorimotor Function

When designing an aerobic exercise training program, one major component is determining the exercise intensity; this becomes even more complex with working with people with DPN. Individuals with diabetes are at greater risk for having a heart attack or stroke due to increases in blood pressure and cholesterol levels [76,77]. In certain cases, those with diabetes are prescribed medications to manage cardiovascular disease, which can blunt certain responses to exercise and limit the intensity of work being done. Moreover, cardiovascular autonomic neuropathy is a common complication with T2D that often goes unrecognized because it presents with non-specific symptoms, such as resting tachycardia and reduced heart rate variability, exercise intolerance, and orthostatic hypotension [78]. This autonomic dysfunction increases the risk for cardiovascular events and mortality several-fold. For these reasons, it is critical to monitor heart rate, blood pressure, and perceived exertion during aerobic exercise training in people with DPN.

Research has supported that moderate to vigorous aerobic exercise prescription using heart rate (HR) metrics, such as percentages of maximum HR (MHR) or HR reserve (HRR), may be an effective method for improving the signs and symptoms of DPN in patients with T2D [35,40,43,44,79]. Dixit et al. [35] had 29 subjects with DPN complete an 8-week intervention of moderate intensity (40–60% HRR) treadmill exercise, 3–6 days per week. The experimental group demonstrated increased nerve conduction velocity (NCV) in both the distal peroneal (42.48 ± 1.25 m/s to 45.56 ± 1.24 m/s) and sural sensory nerves (23.67 ± 1.81 to 31.39 ± 1.58), as well as improvements in mean scores of the Michigan Diabetic Neuropathy Score (12.57 ± 1.74 to 7.03 ± 1.86) [35]. It was suggested that the modulation of sorbitol levels was the potential mechanism for the resulting improvements. Increased intracellular sorbitol concentration has been shown to damage Schwann cells, which can create a chronic hypoxic environment for nerves [35,80,81]. Gholami et al. [43] observed a significant increase in sural sensory NCV (35.2 ± 4.3 m/s to 37.3 ± 6.2 m/s) following 12 weeks of aerobic training with each session lasting 20–45 min at an intensity of 50–70% HRR. Gholami et al. speculated that the improvements in NCV may be associated with greater glycemic control and/or favorable adaptations in vascular components [43].

Hyperglycemia can propagate superoxide production, which can lead to endothelial dysfunction and vascular distensibility [82]. Previous research has demonstrated that aerobic exercise training can improve endothelial function through enhance flow-mediated dilation in people with type 2 diabetes [83,84,85,86,87]. One adaptation to aerobic endurance training may have been the enhanced endothelial function through increased nitric oxide production resulting in improved arterial compliance and a reversal of the hypoxic state created with normal DPN progression [35]. Azizi et al. [44], using 8-week intervention with aerobic exercise intensity at 70–85% MHR, observed sural sensory nerve and tibial compound muscle action potential increased, while F-wave and NCV decreased significantly. Moreover, conduction velocity for the deep peroneal nerve also showed a significant increase. The resulting changes in electrophysiological measures were thought to be a product of decreased lower limb edema, improved blood supply for metabolic demands, and the promotion of neural collateral sprouting, leading to enhanced electrical activities at the specific sites of recording [44].

Neuropathic pain coupled with decreased sensorimotor function may cause individuals with DPN to be physically inactive and therefore an important target for aerobic training interventions. A 16-week supervised aerobic exercise program resulted in a significant reduction in perceived pain (average of 7 pain interference items) during various daily activities (4.29 ± 2.70 to 2.36 ± 2.22) [40]. It was thought to be a function of a decreased level of advanced glycation end products and protein kinase C, which are typically increased due to chronic hyperglycemia. These decreases were suggested to cause a change in central pain processing mechanisms associated with DPN; the appropriate objective measures of brain functioning were not assessed within the study [40]. After completion of a 15-week high-intensity interval training intervention, Hamed and Raoof [38] observed improvements in pain via the Leeds Assessment of Neuropathic Symptoms and Signs Scale (−3.6 ± 0.7 points), in conjunction with significant decreases in fasting (−6.5 ± 2.9 mg/dL) and 2-h (−20.15 ± 2.2) blood glucose. It was suggested that diabetic neuropathic pain could be reduced through exercise by attenuating changes in high and low voltage activated Ca^2+^ channels, thereby delaying the onset of tactile hypersensitivity of the neurons [38].

#### 4.2.2. Physical Fitness

Sedentary behavior has been a longstanding global pandemic and is recognized as one of the top five leading contributors to premature mortality [88,89]. Moreover, an individual’s level of cardiorespiratory fitness is strongly associated with their risk of premature death from all causes and how much physical activity they engage in [90]. Fatigue during common daily activities is a common symptom reported by individuals with type 2 diabetes and DPN, further propagating a sedentary lifestyle potentially resulting in lower cardiorespiratory fitness, decreased muscular strength, and increased body fat [91,92]. Morrison et al. [36] had 16 individuals with DPN complete 12 weeks of aerobic exercise sessions occurring three times per week. Subjects were randomly assigned to one of two isocaloric aerobic exercise training groups: (1) moderate intensity aerobic training (45 min done at 50% of HRR) or (2) vigorous intensity aerobic training (30 min at 75% of HRR). Aerobic exercise during each training session consisted of treadmill walking or running, stationary cycling, and/or elliptical strider workouts done at the correct intensity for the prescribed amount of time on three, nonconsecutive days per week. Following the intervention, only slight increases were observed in peak oxygen uptake ($\overset{\cdot }{V}O$_2peak_) (16.88 ± 1.11 to 17.41 ± 1.42 mL/kg/min); however, significant improvements were seen in fasting blood glucose levels (7.6 ± 0.9 to 7.5 ± 1.1 mmol/l), glycated hemoglobin (Hba1c) (7.3 ± 0.3 to 7.2 ± 0.5%), and insulin (15.9 ± 3.3 to 14.5 ± 3.2 µU/mL) concentrations. Additionally, participants demonstrated reductions in body fat (38.9 ± 1.6 to 37.3 ± 1.8%), increased gait velocity (108.91 ± 1.73 to 116.42 ± 2.15 cm/s), stride length (59.24 ± 0.65 to 62.73 ± 0.81 cm), step length (122.81 ± 1.28 to 125.80 ± 1.59 cm), and a decrease in time spent in stance (63.6 ± 0.9 to 61.8 ± 1.0%) [36]. Kluding et al. [39] prescribed 16 weeks of moderate intensity (50–70% $\overset{\cdot }{V}O$_2reserve_) three times per week. The resulting data displayed a significant increase in $\overset{\cdot }{V}O$_2peak_ (16.2 ± 3.6 to 17.3 ± 4.0 mL/kg/min) and a decrease in total body fat (44.79 ± 6.62 to 43.79 ± 6.64%). Additionally, improvements were found in general (15.7 ± 2.3 to 12.2) and physical (15.5 ± 2.4 to 12.4 ± 3.7) fatigue scores, both collected using the Multidimensional Fatigue Inventory questionnaire [39]. Individuals with diabetes are known to have blunted HR responses during exercise and peripheral vascular dysfunction, which are important contributors to lower exercise capacity, which in turn can limit maximal oxygen consumption [93,94,95]. In conjunction with the increases in aerobic capacity, Kluding et al. [39] observed a significant improvement in flow-mediated dilation at the brachial artery (4.92 ± 3.7 to 7.19 ± 4.39%). Furthermore, high levels of HbA1c are associated with oxidative stress and inflammatory markers, which have been shown to be inversely related to aerobic capacity [96,97]. Based on the evidence provided by the aforementioned studies, there is preliminary evidence that participation in regular aerobic exercise at both moderate and vigorous intensities (% HRR or $\overset{\cdot }{V}O$_2reserve_) for durations of ≥8 weeks can result in favorable improvements in cardiorespiratory fitness and metabolic function, which may prompt greater participation in subsequent daily physical activity and enhance glycemic control.

### 4.3. Resistance Exercise Training

Resistance exercise training is an established method for increasing muscular endurance, size, strength, and power [98,99,100]. For the purpose of this article, resistance training will be defined as “a specialized method of conditioning, which involves the progressive use of a wide range of resistive loads and a variety of training modalities designed to enhance health, fitness, and sports performance” [101]. Though resistance training is a popular form of exercise, its use in clinical trials as the sole intervention tool with individuals with DPN is fairly uncommon. To the best of our knowledge, only one study was identified that fit the criteria of this review [46]. In order to prompt future research in this area, the following subsections will briefly discuss the potential benefits of resistance exercise training on glycemic control, microvascular perfusion, and neural drive, all of which may be key mediators of neuropathic muscle adaptation.

#### 4.3.1. Glycemic Control

Growing evidence demonstrates the efficacy of resistance exercise to promote overall metabolic health in individuals with T2D through improvements in glycosylated hemoglobin (HbA1c) and insulin sensitivity, particularly in the early stages of T2D among those with a lower body mass index [75,102,103]. Participation in regular RT to help manage glucose intolerance and diabetes becomes even more necessary when discussing older adults (i.e., >55 years). With aging, there is a subsequent decline in skeletal muscle mass, often referred to as sarcopenia [104]. This continual decrease in muscle mass leads to the increased risk of developing impaired glucose tolerance and an eventual diagnosis of diabetes since lean-body mass tissue is the primary site of blood glucose uptake [105,106]. Specifically, RT has been shown to decrease glucose area-under-the-curve by 25–28% during oral glucose tolerance tests and increase glucose disappearance rates with subsequent improvements in body composition in older adults [107,108]. When working with individuals with impaired glucose tolerance or diabetes, it is recommended to begin with 1–3 sets of 10–15 repetitions at “moderate” intensities of 50–69% 1-RM for 8–10 exercises targeting the full body. Gradual progression should then be made to sets of 8–10 repetitions corresponding to “vigorous” intensities of 70–85% 1-RM. A review of the literature found that exercise training programs ≥8 weeks with 2–3 sessions per week lasting 30–60 min using progressive RT targeting 5–10 muscle groups seem to provide the greatest benefits. Working sets ranged from 2–6 with 6–20 repetitions of each exercise with the average being 2–3 sets of 8–12 repetitions [75,93,102]. However, it is worth noting that these exercise parameters are derived from work with individuals with diabetes, not DPN, so modifications may be required to suit the specific needs of the participating population.

#### 4.3.2. Microvascular Perfusion

Impaired function of the peripheral vasculature is a common issue that emerges in patients with diabetes, and the development of DPN only further exacerbates the decrease in circulation and perfusion [109]. In a recent systematic review, Garcia-Mateo et al. [110] found that chronic strength training elicited moderate to large (Cohen’s *d* = −1.20 to −1.49) decreases in arterial stiffness measures in healthy individuals. Maeda et al. [111] observed that a 12-week RT program increased nitric oxide production in healthy older men (39.6 ± 3.2 to 61.2 ± 10.4 µmol/L). Theoretically, the increased bioavailability of nitric oxide will elicit greater vasodilation of involved blood vessels allowing for more blood flow and subsequent perfusion of the targeted tissues. Phillips et al. [112] demonstrated that 20 weeks of whole body RT increased leg blood flow and vascular conductance in various feed states among young (18–28 year), middle aged (45–55 year), and older (65–75 year) adults. Finally, Cook et al. [113] demonstrated that a 6-week RT intervention significantly lowered biomarkers indicative of endothelial function (matrix metalloprotease; 276.3 ± 31.6 to 171.1 ± 18.3 ng mL^−1^) and oxidative stress (8-Isoprostane; 349.8 ± 28.9 to 299.8 ± 34.9 pg mL^−1^) in African American men. Though the discussed data was not collected in patients with DPN, it does suggest that RT may be effective in stimulating favorable vascular changes and improving endothelial function in this population. Though the aforementioned results were observed in a healthy population, it gives insight into the potential effects possible from chronic RT. Future research should attempt to replicate or build on the current studies, limiting enrollment to those with DPN in order to investigate whether similar or even greater improvements in circulation and perfusion can be attained.

#### 4.3.3. Neural Drive

Individuals with DPN are at an increased risk of falling relative to age-matched controls, with 60% of fall-related deaths occurring during descent down stairs [46,114,115,116]. Previous research has shown that DPN patients are slower to develop ankle and knee joint force, which has been attributed to diminished muscle activation [46,117,118,119,120]. This decrement in strength and power seen in patients with DPN can be further exacerbated in older adults who contend with age-related sarcopenia [14,121]. Engagement in regular RT has been demonstrated to be a primary intervention for increasing strength and power through neural adaptations across ages [122,123,124]. The use of heavy external loads, high velocity movements, and motor skill learning within RT aids in the increase of efferent neural drive through improved motor unit recruitment, motor neuron firing frequencies, motor unit synchronization, and the attenuation of antagonist co-activation [14,125]. With these neuromuscular adaptations in mind, various RT programs have been utilized to help reduce fall risk through enhanced balance and stability, increased core strength, work efficiency, and skeletal muscle fiber size [126,127,128]. Liu-Ambrose et al. [126] saw a 57.3% reduction in Physiological Profile Assessment fall-risk scores and a 30.6% reduction in postural sway in community-dwelling older women following resistance training. However, RT focused interventions conducted in patients with DPN are lacking within the scientific literature. Handsaker et al. [46] had participants engage in a 16-week RT intervention. Training sessions involved both dynamic and isometric exercise. Participants performed of 3 sets of 12 repetitions of leg extension, leg press, and ankle press with 1-min rest periods between sets. Dynamic exercise loads were auto-regulated to maintain progressive overload throughout. Post-testing results demonstrated that speed of strength generation in the ankle and knee was significantly higher in subjects with DPN during both stair ascent and descent.

### 4.4. Concurrent Aerobic and Resistance Exercise Training

As previously mentioned, aerobic exercise is the most commonly employed training intervention in the scientific literature investigating exercise effects on people with DPN. Conversely, research looking into the efficacy of resistance exercise training is lacking. However, a concurrent aerobic and resistance exercise training approach has been studied in people with DPN.

#### Sensorimotor Function

Kluding et al. [47] had 17 individuals with DPN participate in a 10-week aerobic and strengthening exercise program with supervised sessions taking place 3–4 days per week. The intervention resulted in a significant reduction in pain via a visual analog scale (mean ± standard deviation; 62.4 ± 26.7 to 44.3 ± 35.1), a Michigan Neuropathy Screening Instrument symptom score (5.24 ± 1.4 to 4 ± 2), and an increase in intraepidermal nerve fiber branching in proximal lower extremity skin biopsies (0.16 ± 0.15 to 0.27 ± 0.19) [47]. Nadi et al. [49] also observed improvements in sensory-motor function after utilizing combined training. Forty-two individuals with DPN experienced significant reductions in numbness, pain, tingling, and weakness in the lower limbs and increases in sense of touch intervention, detection of finger position, and vibration perception in tissues. However, it should be noted that exercise training was done over 12 weeks in conjunction with vitamin D supplementation [49]. Stubbs Jr. et al. [50] found non-significant alterations in peripheral sensory and motor nerve electrophysiologic properties when comparing the effects of 12-week aerobic, isokinetic strength, and combined aerobic and isokinetic strength exercise training interventions.

It has been speculated that the length of the intervention and the degree of damage sustained by the peripheral nerve fibers play a major role, with shorter interventions (e.g., ≤10 weeks) affecting less extensively damaged fibers while longer interventions are necessary when damage is more severe [47]. One explanation given for the reversal of neuropathy signs and symptoms following exercise could be the increased ability for vasodilation in blood vessels allowing for greater blood flow and perfusion of peripheral nerves. Proteins carried within the blood prompt the recovery of myelin and an increase in neural axons [49]. The eventual increase in axon connections are hypothesized to improve neural function in people with DPN. Implementing a concurrent aerobic and resistance training program may allow for compounding improvements in DPN signs and symptoms; however, conflicting evidence still exists in the literature. More research is needed with future studies focused on exclusively utilizing long-term (i.e., >12 weeks) exercise-based interventions with large sample sizes and a comprehensive battery of outcome testing measures to gauge nerve conduction, fiber density, sensory-motor function, and physical function. Additionally, more studies need to be dedicated toward investigating the efficacy of using RT interventions alone, for improving microvascular perfusion and neural drive within individuals with DPN.

### 4.5. Balance and Proprioception-Based Exercise Training

Damage to the sensory and motor nerves can severely impair myofiber innervation and perfusion, causing muscle atrophy and balance impairments, which is of even greater concern for older adults already at an elevated risk of developing sarcopenia and falling [8,9,10]. For these reasons, practitioners have sought to implement therapeutic strategies focused on improving balance.

Song et al. [52] had 19 individuals with DPN participate in an 8-week balance exercise program, two times a week for 50 min per session. Results from the study demonstrated that body sway significantly decreased during (1) eyes open anterior–posterior (45.9 ± 12.3 to 33.9 ± 7.9 cm), medial–lateral (50.4 ± 29.3 to 33.2 ± 6.9 cm), and total body sway (76.1 ± 32.4 to 52.3 ± 11.5 cm) and (2) eyes closed anterior–posterior (62.0 ± 19.9 to 49.3 ± 13.6 cm), medial–lateral (58.8 ± 27.1 to 49.9 ± 16.8 cm), and total body sway (94.9 ± 36.8 to 79.3 ± 25.6 cm). The one-leg stance test significantly increased for eyes open (left: 6.2 ± 4.0 to 9.9 ± 8.3 s, right: 5.9 ± 5.2 to 11.6 ± 7.0 s), eyes closed (left: 3.2 ± 2.1 to 4.8 ± 2.1 s, right: 3.3 ± 2.3 to 5.7 ± 2.3 s), and head rotation (left: 3.5 ± 2.4 to 5.4 ± 2.7 s, right: 4.2 ± 2.6 to 7.6 ± 5.4 s). Song et al. [52] also observed improvements in dynamic balance score from the Berg Balance Scale (53.0 ± 2.3 to 55.1 ± 1.1), Functional Reach Test (27.1 ± 7.4 to 30.9 ± 6.1 cm), Timed Up and Go test (11.8 ± 2.3 to 10.1 ± 2.1 s), and 10-m walking time (9.6 ± 1.4 to 8.7 ± 1.2 s) following balance exercise training. Finally, favorable changes in trunk proprioception were seen through a decrease in trunk repositioning errors across three scenarios: eyes open stable surface (1.6 ± 1.1 to 0.9 ± 0.8), eyes closed stable surface (1.8 ± 1.2 to 0.7 ± 0.5), and eyes open foam (1.9 ± 1.3 to 1.1 ± 0.6) [52].

The postural instability observed in individuals with DPN seems to be a result of inaccurate proprioceptive feedback from the lower extremities. Decreases in proprioception results in the loss of the ability to adequately coordinate essential reflexes and joint movements, in addition to complex balance and postural control [129,130]. Grewal et al. [61] had 18 individuals with DPN complete 4-week sensor-based balance training. Compared with the control group, the subjects in the experimental group showed a significantly reduced center of mass sway (3.67 ± 2.99 to 1.53 ± 1.44 cm^2^; 58.31%), ankle sway (1.85 ± 1.82 to 0.69 ± 0.60 degree^2^; 62.7%) and hip joint sway (2.50 ± 4.65 to 0.69 ± 0.77 degree^2^; 72.4%) during the balance test with open eyes. Only the ankle sway was significantly reduced in the experimental group (3.01 ± 3.72 to 1.24 ± 1.13 degree^2^; 58.8%) during measurements while the eyes were closed. Additionally, the number of steps walked showed a substantial but non-significant increase (8656 ± 4589 to 11052 ± 5365; 27.68%; *p* = 0.064) in the experimental group following training [61]. Finally, Hung et al. [63] had 24 individuals with DPN participate in a 6-week interactive video game-based exercise program to improve balance. The interactive video game-based protocol had participants perform 30-min training sessions consisting of tasks designed to enhance lower body strength, balance, and coordination. Study participants had improvements in the Berg Balance Scale score (48.92 ± 1.29 to 52.33 ± 1.01), Unipedal Stance Test with eyes open (right: 10.52 ± 3.07 to 18.89 ± 3.93 s; left: 10.05 ± 2.18 to 22.71 ± 4.86 s), and the Timed Up and Go and Modified Falls Efficacy Scale (115.25 ± 9.06 to 125.83 ± 6.36) [63], suggesting a decreased risk of falling.

Yoga-based interventions have been suggested as an alternative to traditional balance and flexibility exercise therapy. Boslego et al. [57] found that yoga significantly improved Berg Balance Scale scores (49.33 ± 6.43 to 53.00 ± 4.68) and increased balance confidence scores (68.96 ± 18.41 to 76.10 ± 17.38) in individuals with DPN, even more so than conventional balance exercises. Kanjirathingal et al. [58] investigated a 12-week yoga training intervention and also observed significant increases in balance and strength outcome measures. The Star excursion balance test resulted in greater reach distances anteriorly (right: 68.1 ± 3.4 to 80.1 ± 5.4 cm; left: 67.5 ± 3.6 to 80.6 ± 5.2 cm), postero-laterally (right: 68.1 ± 2.0 to 79.2 ± 4.0 cm; left: (67.6 ± 3.4 to 79.5 ± 4.2 cm), and postero-medially (right: 59.3 ± 3.3 to 67.3 ± 3.1 cm; left: 59.3 ± 3.4 to 67.2 ± 3.5 cm). Increases were also demonstrated in single limb stance time for both eyes closed (right: 3.5 ± 1.4 to 11.0 ± 3.2 s; left: 3.6 ± 1.3 to 11.4 ± 3.4 s) and eyes open (right: 17.7 ± 4.0 to 49.7 ± 22.5 s; 17.4 ± 4.3 to 50.3 ± 22.1 s). Finally, Kanjirathingal et al. [58] found yoga training increased lower extremity strength during a chair stand test (9.0 ± 1.2 to 12 ± 1.8 reps) and a step-up test front facing (8.0 ± 1.2 to 11.3 ± 1.4 reps), laterally right (8.0 ± 1.5 to 10.2 ± 2.7 reps), and laterally left (9.0 ± 1.4 to 10.7 ± 2.7 reps). Though both the yoga training and the conventional balance exercises demonstrated effectiveness in boosting balance and strength performance, the yoga intervention was concluded to be marginally better for improvements in both outcome categories compared to conventional balance training [58].

Another modality of exercise training practitioners have investigated is the use of tai chi, or tai chi chuan. Tai chi is a Chinese-based martial arts dating back more than 3000 years. In the modern day, tai chi is utilized as a form of exercise characterized by self-paced, well-controlled physical movements synced with focused breathing, stretching, and relaxation techniques [131]. In addition to being a popular form of physical activity and meditation, evidence exists indicating its efficacy for helping to treat adults with various chronic physical and metal conditions [132,133,134,135,136,137,138,139]. This form of physical activity is best-known for its efficacy with improving balance and postural control [140,141,142,143]. Ahn and Song [59] had 20 individuals with DPN participate in a 1-h tai chi session, two times per week for 12 weeks. Improvements were observed in glucose control (fasting blood glucose: 137.8 ± 45.2 to 125.5 ± 45.6 mg/dL), balance score (22.37 ± 23.7 to 30.0 ± 28.1), and neuropathic total symptom score (1.1 ± 2.0 to 2.0 ± 1.9) [59]. Though this form of exercise shows some promise as a viable modality for treating patients with DPN, particularly older adults, more research is needed.

### 4.6. Whole-Body Vibration Exercise Training

A primary characteristic of DPN is chronic pain caused by accumulated damage to the afferent nociceptors from the chronic hyperglycemia [144]. This damage leads to hyper-excitation of the central nerves and the spontaneous generation of nerve impulses throughout the periphery [145]. A significant decrease in balance and mobility, coupled with chronic pain, result and worsen as time goes on. Treatments for DPN, specifically pathology affecting sensorimotor function, focus on symptom management through pharmacological interventions because there are no effective therapeutics that target the underlying neuropathies. Whole-body vibration training (WBVT) has been studied as an intervention for DPN pain management. WBVT most commonly involves individuals exercising on a vibrating platform and has been proposed as an alternative to traditional strength training. The vibrations created from the platform mechanically generate rapid changes in the muscle-tendon complex length, which, in turn, stimulates repetitive, reflexive contractions of the muscle, subsequently leading to increased muscular fitness [146]. WBVT has been shown to have favorable effects on muscle function, flexibility, oxygen uptake, body composition, and blood pressure [147,148,149,150,151]. Unfortunately, the research looking into the efficacy of vibration therapy among people with DPN is limited. 

Kessler and Hong [65] found that 4 weeks of vibration treatment sessions, occurring three times per week, resulted in acute pain reduction in DPN patients using both visual analog and neuropathic pain scales. A follow up study from Kessler et al. [67] found similar decreases in pain over both a 2- and 4-week interval. Finally, Lee et al. [66] conducted a 6-week whole body vibration therapy intervention in elderly patients with DPN. Following the WBVT, balance improved during eyes open (anterior–posterior: −4.05 ± 2.09 mm/s; medial–lateral: −2.36 ± 1.80 mm/s; sway velocity-movement: −6.90 ± 3.82 mm/s^2^) and eyes closed (anterior–posterior: −5.74 ± 2.53 mm/s; medial–lateral: −3.72 ± 3.02; sway velocity-movement: −9.30 ± 7.29 mm/s^2^) sway velocity testing. One leg stance time significantly increased for eyes open (6.41 ± 4.59 s) and closed (2.09 ± 1.30 s). Lee et al. [66] also observed increased Berg Balance Scale scores (1.89 ± 1.52), Functional Reach Test (4.45 ± 3.52 cm), Timed Up and Go test (−1.79 ± 1.09 s), and the Sit-to-Stand test (−3.68 ± 2.40 s). Though promising, further research is needed to explore the efficacy of vibration therapy for treatment of DPN. Though the results of the aforementioned studies seem promising, it has been shown that long duration exposure to WBV at high frequencies can have dangerous side effects, including increased lumbar spinal degeneration, lower back pain, muscular fatigue, and contributions to the pathogenesis of disorders in female reproductive organs [152,153,154,155]. Therefore, more research is needed to evaluate the efficacy of WBV in people with DPN, as well as to develop safe exercise protocols.

## 5. Conclusions

Upon reviewing the literature, there is support that exercise-based interventions ≥4 weeks are beneficial for patients with DPN. This conclusion falls in line with other recently conducted literature reviews investigating the effects of exercise on enhancing gait function [156], decreasing neuropathic pain [157], and improving posture and balance among patients with peripheral neuropathy [158]. However, though the results of this review find various modalities of exercise to be beneficial, the effects are quite numerous and at times, inconsistent. Mobility and functional movement-based training, specifically weight-bearing exercise, is not only safe for people with DPN to participate in, but can provide significant increases in gait, balance, and strength. Of the six studies reviewed in the current paper, the overlapping result seems to be the enhancement of lower-body movement. The training programs implemented observed improvements in gait velocity, cadence, ankle joint mobility, and decreased step time. There were also favorable changes in plantar pressure and other kinematic and kinetic variables recorded during gait analyses. Improvements were thought to be tied to increases in foot and ankle musculature strength, as well as greater coordination of muscle activation, body stability, and joint flexibility. From a practical perspective, following weight-bearing exercise training, DPN patients demonstrated greater daily step counts and walking distances. From a clinical perspective, all these results lend themselves well towards improving daily physical function and quality of life for patients with DPN. For practical application, the research suggests that exercise sessions should start using “light” intensities and then slowly titrate up over time. Moreover, non-weight bearing mobility and functional training can take place first and as patients show improvement, they can be transitioned to weight-bearing exercise.

As previously stated, aerobic exercise is one of the most commonly utilized training modalities in both healthy and special populations. Aerobic exercise training at moderate intensities (40–70% of HRR or $\overset{\cdot }{V}O$_2reserve_) has shown the ability to improve sensorimotor function, cardiorespiratory endurance, reduce fatigue, and promote increased physical activity. Mechanisms for the observed improvements following aerobic exercise interventions may be tied to enhanced glycemic control and endothelial function. Furthermore, three studies demonstrated improvements in electrophysiological examinations, specifically in greater nerve conduction velocity within the sensory nerves [35,43,44]. These significant changes are also thought to be influenced by changes in glycemic status, as well as decreases in lower limb edema and increases in blood supply to meet certain metabolic requirements for neural collateral sprouting. Because NCS are regarded as the standard for measuring peripheral nerve functions, clinicians should look to incorporate aerobic exercise into treatment protocols for patients with DPN, as a non-pharmacological aide to help bolster the effects of prescribed medications and other therapy.

Resistance exercise training has many potential benefits for individuals with DPN and has demonstrated its effectiveness when combined with aerobic exercise. Training programs should be designed utilizing 1–3 sets of 10–15 repetitions at “moderate” intensities of 50–69% 1-RM for either 2–4 lower-body focused exercises or 8–10 exercises targeting the full body to elicit potential favorable changes in glycemic control, microvascular perfusion, and neural drive. However, more research is needed with resistance exercise training being the sole intervention modality being utilized to gain a better understanding of how it functions independently.

Traditional balance, proprioception-based, yoga, and tai chi-based exercise training interventions have all demonstrated the ability to improve multiple different measures of static and dynamic balance performance, postural control, and strength, and to reduce fall risk. For individuals who are less physically fit, older, or have more severe cases of peripheral neuropathy, these modalities of exercise may be a more efficacious choice. Finally, WBVT shows promise as both an alternative to conventional strength training and a viable intervention method for increasing motor function in subjects with DPN. However, similar to traditional resistance exercise training, more research is needed to fill the current gap in the scientific literature and determine the safest manner in which to implement WBV in the long term.

Due to the wide range and variability in observed results found in the current literature review, the authors recommend that prior to selecting an exercise-based intervention to implement in individuals with DPN, practitioners should perform a needs assessment of their patient(s) and determine a priority list in order to decide the most appropriate modality to choose. Future studies should look to perform more multi-group studies comparing various exercise training modalities against one another and combined. Furthermore, comprehensive outcome assessments need to be implemented to specify which type of training demonstrated the largest effects for a particular test. Based on the findings of the current review, the authors recommend that outcome assessments include NCS, neuropathic signs/symptoms assessments, performance tests to evaluate physical function as it relates to lower-body strength, gait, balance, and posture control, and analyses of metabolic biomarkers specifically related to glycemic control.

## Figures and Tables

**Table 1:** Summary of exercise training intervention studies in individuals with diabetic peripheral neuropathy.

Author (Year)	Participants	Primary Measures	Intervention					Key Results
			Type	Frequency	Time	Intensity	Duration	
Mueller (2013) [8]	Weight-bearing (15); non-weight-bearing (14)	6-min walk and daily step count	Mobility and functional movement	3 days/week	60 min/session	60–70% MHR	12 weeks	Weight-bearing group: greater improvements in 6-min walk and daily step count; non-weight-bearing group: greater improvements in HbA1c.
El-Refay and Ali (2014) [30]	Control (15); experimental (15)	Gait	Mobility and functional movement	3 days/week	45–60 min/session	---	8 weeks	Increased walking speed, cadence, and ankle ROM; decreased step time
Sartor (2014) [10]	Control (29); experimental (26)	Gait	Mobility and functional movement	2 days/week	40–60 min/session	---	12 weeks	No significant change in foot rollover during gait
Kanchanasamut and Pensri (2017) [31]	Control (10); experimental (11)	Foot mobility, plantar pressure, and foot sensation	Mobility and functional movement	5 days/week	---	---	8 weeks	Increased ROM and decrease peak plantar pressure
Win (2020) [32]	Control (53); experimental (51)	Activities of daily living, DPN signs/symptoms, and pain	Mobility and functional movement	3 sessions/day; 2–3 days/week	10 min/session	---	8 weeks	Improvements in motor scores and activities of daily living
Monteiro (2020) [33]	Control (15); experimental (15)	Strength, PA, gait speed, ROM, DPN symptoms, and QOL	Mobility and functional movement	2 days/week	50 min/session	---	12 weeks	Improvements in toe strength, gait, DPM symptoms, and foot contact pressure
Dixit (2014) [34]	Control (37); experimental (29)	Neuropathy quality of life	Aerobic	5–6 days/week	150–360 min/week	40–60% HRR	8 weeks	Improved neuropathy quality of life total score
Dixit (2014) [35]	Control (37); experimental (29)	Nerve conduction studies and MDNS	Aerobic	3–6 days/week	150–360 min/week	40–60% HRR	8 weeks	MDNS scores decreased and NCV increased
Morrison (2014) [36]	Non-DPN (21); DPN (16)	Gait, reactions, fall risk, and balance	Aerobic	3 days/week	30–45 min/session	50–75% HRR	12 weeks	Reaction time decreased, gait velocity and stride/step length increased, balance and postural coordination improved
Zhang (2014) [37]	Control (30); experimental (30)	Plantar pressure	Aerobic	3 days/week	20–40 min/session	100–120 bpm	12 weeks	Peak plantar pressure in forefoot decreased while pressure in the medial foot increased
Hamed (2014) [38]	DPN (40); HIIT group (20); aerobic group (20)	Leeds Assessment of Neuropathic Symptoms/Signs Scale	Aerobic	3 days/week	Aerobic: 50 min; HIIT: 20 min	Aerobic: 50–60% MHR; HIIT: 85–95% MHR	15 weeks	HIIT lead to greater reductions in pain outcome compared to moderate aerobic exercise
Kluding (2015) [39]	Experimental (18)	Adverse events, fatigue, and $\overset{\cdot }{V}O$_2peak_	Aerobic	3 days/week	30–50 min/session	50–70% $\overset{\cdot }{V}O$_2reserve_	16 weeks	57 nonserious adverse events occurred and improvements occurred in general fatigue, physical fatigue, and $\overset{\cdot }{V}O$_2peak_
Yoo (2015) [40]	Experimental (14)	Pain intensity and pain interference	Aerobic	3 days/week	30–50 min/session	50–70% $\overset{\cdot }{V}O$_2reserve_	16 weeks	Pain interference was reduced in walking, normal work, relationship with others, and sleep
Dixit (2016) [41]	Control (36); experimental (28)	Balance and posture stability	Aerobic	3–6 days/week	150–360 min/week	40–60% HRR	8 weeks	Moderate improvement on eyes closed sway velocity on foam
Billinger (2017) [42]	experimental (17)	FMD	Aerobic	3 days/week	30–60 min/session	50–70% $\overset{\cdot }{V}O$_2reserve_	16 weeks	Improvements in peak diameter and time to peak shear, but not statistically significant
Gholami (2018) [43]	Control (12); experimental (12)	Nerve conduction studies	Aerobic	3 days/week	20–45 min/session	50–70% HRR	12 weeks	NCV increased but potential amplitude was not different from control
Azizi (2019) [44]	Experimental (35)	Nerve conduction studies	Aerobic	3 days/week	40–45 min/session	70–85% MHR	8 weeks	Improvements in both action potential amplitude and conduction velocity
Gholami (2020) [45]	Control (15); experimental (16)	FMD, IMT, vessel diameter, and MDNS	Aerobic	3 days/week	30–45 min/session	50–70% HRR	12 weeks	Significant improvements in FMD and MDNS
Handsaker (2016) [46]	Control (21); non-DPN (13); DPN (9)	Speed of ankle and knee strength generation	Resistance training	1 day/week	60 min/session	12 RM	16 weeks	Ankle and knee speed of strength generation were higher in both stair ascent and descent
Kluding (2012) [47]	Experimental (17)	Pain, MNSI, nerve function, and intraepidermal nerve fiber	Aerobic and resistance training	3–4 days/week	30–50 min/session (aerobic)	50–70% $\overset{\cdot }{V}O$_2reserve_; 7–8 RPE	10 weeks	Reduction in pain, neuropathic symptoms, and increased intraepidermal nerve fiber branching
Taveggia (2014) [48]	Control (14); experimental (13)	6-min walk and 10-m walking test	Aerobic and resistance training	5 days/week	60 min/session	---	4 weeks	Increased 6-min walk distance
Nadi (2017) [49]	Control (41); experimental (42)	MNSI	Aerobic and resistance training	3 days/week	20–60 min/session	50–70% MHR; 50% 10RM	12 weeks	Reduction in numbness, pain, tingling, weakness; increases in sense of touch
Stubbs (2019) [50]	Control (12); experimental (33)	NCS	Aerobic and resistance training	3 days/week	>30 min/session	60–80% $\overset{\cdot }{V}O$_2peak_	12 weeks	No alterations in sensory/motor nerve electrodiagnostic
Seyedizadeh (2020) [51]	Control (10); experimental (12)	Serum kinesin-1 and physical function	Aerobic and resistance training	3 days/week	≥60 min	8–12 RM; 50–65% HRR	8 weeks	Serum kinesin-1 and aerobic endurance decreased and upper body strength increased (all non-significant)
Song (2011) [52]	Control (19); experimental (19)	Static/dynamic balance and trunk proprioception	Balance	2 days/week	60 min/session	---	8 weeks	Postural sway decreased, one-leg stance increased, dynamic balance improved, and trunk repositioning errors decreased
LeMaster (2008) [53]	Control (38); experimental (41)	Activity level	Balance	3 days/week	60 min/session	---	12 months	Increase in total daily steps
Allet (2010) [54]	Control (35); experimental (36)	Gait	Balance	2 days/week	60 min/session	---	12 weeks	Increased habitual walking speed; improved cadence, gait cycle time, and stance time
Kruse (2010) [9]	Control (38); experimental (41)	Strength, balance, and falls	Balance	3 days/week	60 min/session	---	12 months	Small time increase in 1-leg, eyes closed stand
Eftekhar-Sadat (2015) [55]	Control (17); experimental (17)	TUG, BBS, fall risk, and postural stability	Balance	3 days/week	---	---	4 weeks	Decrease in TUG, fall risk index, and increase overall stability index
Ahmed (2018) [56]	Control (15); experimental (45)	Posture stability	Balance	3 days/week	60 min/session	---	6 weeks	Increased posture stability
Boslego (2017) [57]	Experimental (15)	BBS, balance confidence, and occupation performance/satisfaction	Yoga	2 days/week	60 min/session	---	8 weeks	Improvements in BBS, balance confidence, and occupation performance/satisfaction
Kanjirathingal (2021) [58]	Yoga (11); conventional (10); control (14)	Balance, center of pressure, chair stand, and step-up test	Yoga	3 days/week	60 min/session	---	12 weeks	Improvements in balance, center of pressure, chair stand, and step-up test
Ahn and Song (2012) [59]	Control (19); experimental (20)	Glucose control, neuropathy score, balance, and quality of life	Tai-chi	2 days/week	60 min/session	---	12 weeks	Improved glucose control, balance, neuropathic symptoms, and quality of life
Handsaker (2019) [60]	Control (7); experimental (24)	Stepping accuracy	Proprioception	1 day/week	60 min/session	---	16 weeks	Increased stepping accuracy
Grewal (2015) [61]	Control (16); experimental (18)	Posture stability and daily physical activity	Proprioception	2 days/week	45 min/session	---	4 weeks	Reduced center of mass, ankle, and hip joint sway
Ahmad (2019) [62]	Control (17); experimental (20)	Balance and proprioception	Proprioception	3 days/week	50–60 min/session	---	8 weeks	Increased one-leg stance, decreased TUG, center of pressure sway, and increased proprioception
Hung (2019) [63]	DPN-group A (12); DPN-group B (12)	MFES, TUG, BBS, and UST	Proprioception	3 days/week	30 min/session	---	6 weeks	Improvements occurred in BBS, right-leg UST, and TUG test scores
Rehab and Saleh (2019) [64]	Control (15); experimental (15)	Gait and risk of falling	Proprioception	3 days/week	30 min/session	---	8 weeks	Increased step length, velocity and cadence; decreased risk of falling
Kessler (2013) [65]	Experimental (8)	Neuropathic pain scale and visual analog pain scale	Whole body vibration	3 days/week	12 min/session	25 Hz and 5 mm amplitude	4 weeks	Reductions in both pain scales
Lee (2013) [66]	WBV/balance (19); balance (18); control (18)	Balance, muscle strength, and HbA1c	Whole body vibration	2 days/week (balance); 3×3 min/day	60 min/session	---	6 weeks	Combined vibration and balance training improved static balance, muscle strength, and HbA1c
Kessler (2020) [67]	Control (8); experimental (12)	Visual analog pain scale	Whole body vibration	3 days/week	12 min/session	25 Hz and 0.5–1.0 g	4 weeks	Significant reductions in pain after 2 and 4 weeks

BBS = Berg Balance Scale; DPN = diabetic peripheral neuropathy; FMD = flow-mediated dilation; HbA1c = glycated hemoglobin; HIIT = high-intensity interval training; HRR = heart rate recovery; IMT = intima media thickness; MDNS = Michigan Diabetic Neuropathy Screen; MFES = modified falls efficacy scale; MHR = maximum heart rate; MNSI = Michigan Neuropathy Screening Instrument; NCV = nerve conduction velocity; PA = physical activity; QOL = quality of life; ROM = range of motion; RPE = ratings of perceived exertion; TUG = Timed Up And Go; UST = unipedal stance test; $\overset{\cdot }{V}O$_2_ = oxygen uptake.

## Data Availability

No new data were created or analyzed in this study. Data sharing is not applicable to this article.
